# 5-(3,5-Di­fluoro­phen­yl)-1-(4-fluoro­phen­yl)-3-tri­fluoro­methyl-1*H*-pyrazole

**DOI:** 10.1107/S1600536813032650

**Published:** 2013-12-07

**Authors:** Karikere Ekanna Manoj Kumar, Parameshwar Adimoole Suchetan, Bandrehalli Siddagangaiah Palakshamurthy, Shankar Madan Kumar, Neratur Krishnappagowda Lokanath, Swamy Sreenivasa

**Affiliations:** a Department of Studies and Research in Chemistry, U.C.S., Tumkur University, Tumkur, Karnataka 572 103, India; b Department of Studies and Research in Physics, U.C.S., Tumkur University, Tumkur, Karnataka 572 103, India; c Department of Studies in Physics, University of Mysore, Manasagangotri, Mysore, India

## Abstract

In the title compound, C_16_H_8_F_6_N_2_, the dihedral angle between the pyrazole and di­fluoro­benzene rings is 50.30 (13)°, while those between the pyrazole and fluoro­benzene rings and between the di­fluoro­benzene and fluoro­benzene rings are 38.56 (13) and 53.50 (11)°, respectively. Aromatic π–π stacking inter­actions between adjacent di­fluoro­benzene rings [centroid–centroid separation = 3.6082 (11) Å] link the mol­ecules into dimers parallel to [21-2].

## Related literature   

For background to pyrazole derivatives and their uses, see: Ramaiah *et al.* (1999 ▶). For a similar structure, see: Sreenivasa *et al.* (2013 ▶).
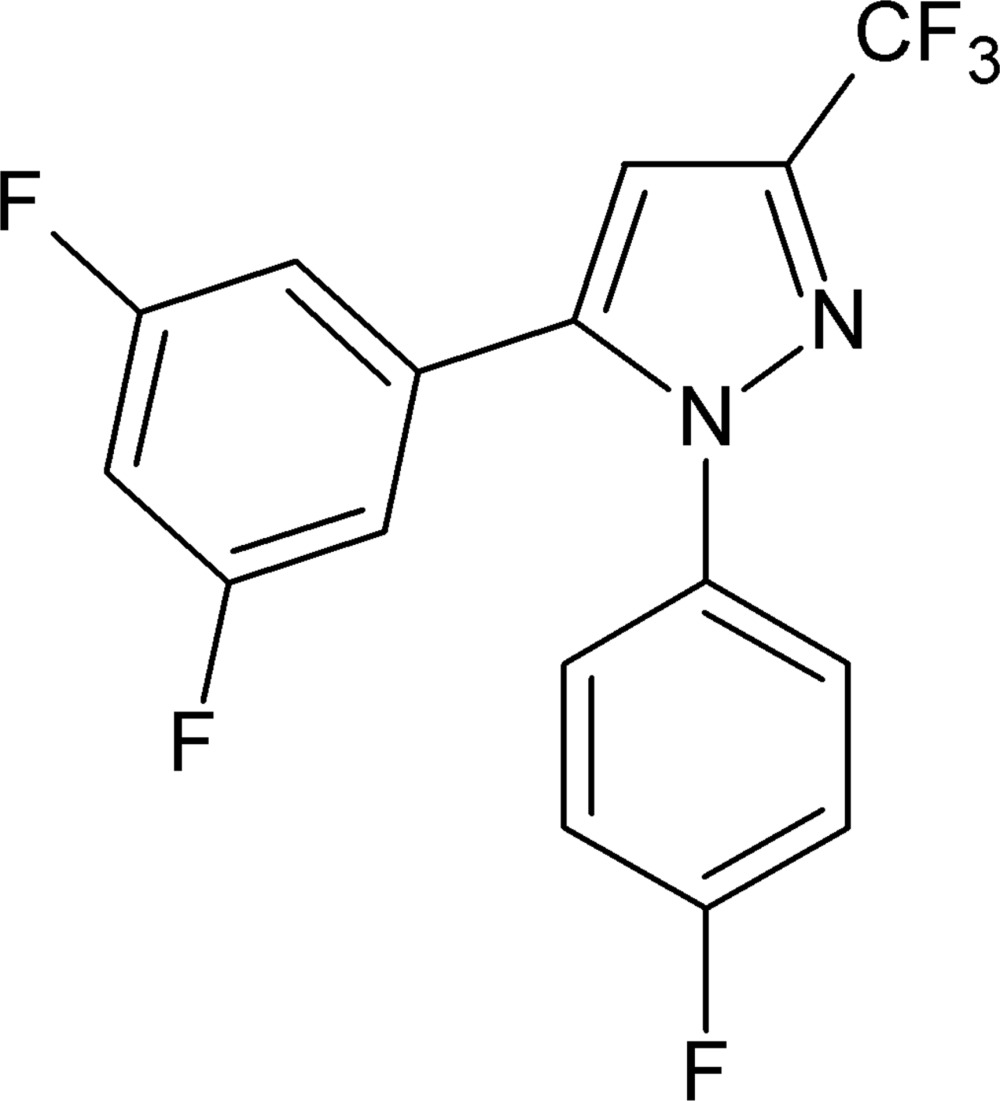


## Experimental   

### 

#### Crystal data  

C_16_H_8_F_6_N_2_
*M* *_r_* = 342.24
Triclinic, 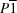

*a* = 7.2535 (3) Å
*b* = 8.6686 (4) Å
*c* = 11.7690 (5) Å
α = 70.909 (1)°
β = 80.139 (1)°
γ = 88.077 (1)°
*V* = 688.78 (5) Å^3^
*Z* = 2
Cu *K*α radiation
μ = 1.39 mm^−1^
*T* = 293 K
0.39 × 0.35 × 0.29 mm

#### Data collection  

Bruker APEXII CCD diffractometer
Absorption correction: multi-scan (*SADABS*; Bruker, 2009 ▶) *T*_min_ = 0.611, *T*_max_ = 0.669
7069 measured reflections
2181 independent reflections
2040 reflections with *I* > 2σ(*I*)
*R*_int_ = 0.042

#### Refinement  

*R*[*F* ^2^ > 2σ(*F* ^2^)] = 0.054
*wR*(*F* ^2^) = 0.181
*S* = 1.11
2181 reflections
217 parameters
H-atom parameters constrained
Δρ_max_ = 0.45 e Å^−3^
Δρ_min_ = −0.48 e Å^−3^

### 

Data collection: *APEX2* (Bruker, 2009 ▶); cell refinement: *SAINT-Plus* (Bruker, 2009 ▶); data reduction: *SAINT-Plus*; program(s) used to solve structure: *SHELXS97* (Sheldrick, 2008 ▶); program(s) used to refine structure: *SHELXL97* (Sheldrick, 2008 ▶); molecular graphics: *Mercury* (Macrae *et al.*, 2008 ▶); software used to prepare material for publication: *SHELXL97*.

## Supplementary Material

Crystal structure: contains datablock(s) I, New_Global_Publ_Block. DOI: 10.1107/S1600536813032650/wm2788sup1.cif

Structure factors: contains datablock(s) I. DOI: 10.1107/S1600536813032650/wm2788Isup2.hkl

Supporting information file. DOI: 10.1107/S1600536813032650/wm2788Isup3.cml

Additional supporting information:  crystallographic information; 3D view; checkCIF report

## Figures and Tables

**Fig. 1.**
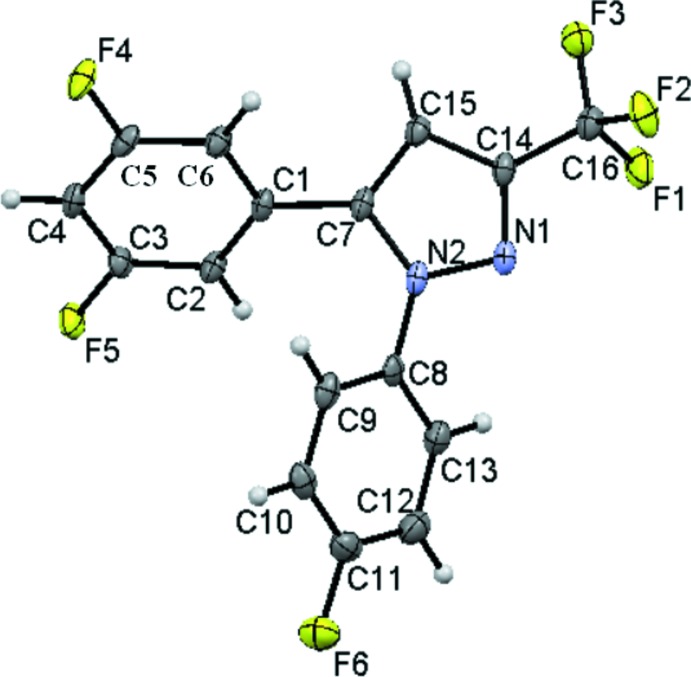
The molecular structure of the title compound, showing the atom-labeling scheme. Displacement ellipsoids are drawn at the 50% probability level.

**Fig. 2.**
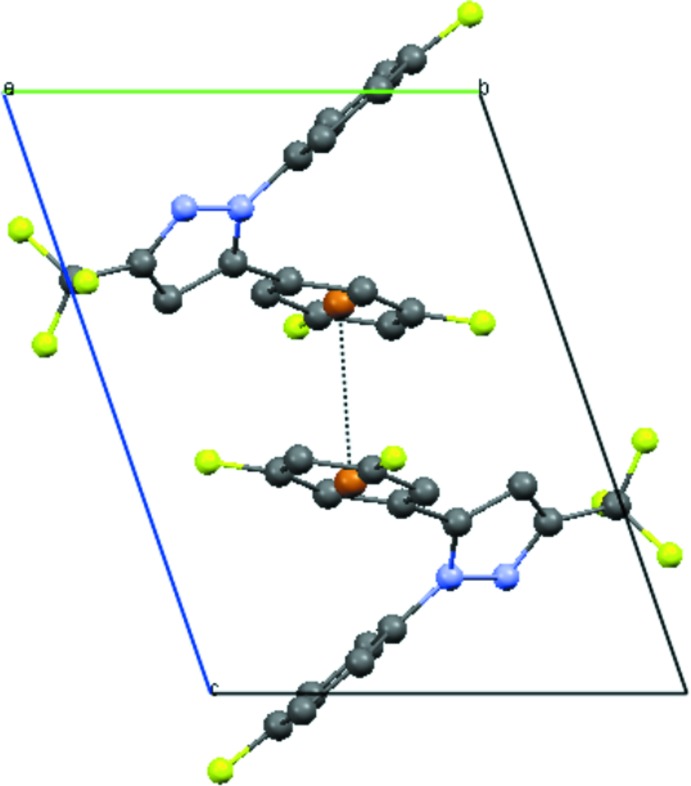
Aromatic Cg···Cg stacking interactions observed in the crystal structure of the title compound. [Cg is the centroid of the difluorobenzene ring. H-atoms are omitted for clarity.]

## supplementary crystallographic information

### 1. Comment

The pyrazole entity is an important moiety in numerous natural and synthetic compounds and in medicinal chemistry (see, for example: Ramaiah et al., 1999). As part of our studies in this area, the title compound, C_16_H_8_F_6_N_2_, was synthesized and its crystal structure determined.

In the title compound, the dihedral angle between the pyrazole and the difluorobenzene rings is 50.30 (13)°, while those between the pyrazole and the fluorobenzene rings and the difluorobenzene and the fluorobenzene rings are, respectively, 38.56 (13)° and 53.50 (11)°. Compared to these values, the dihedral angles between the pyrazole-benzoic acid ring, pyrazole-fluorobenzene ring and fluorobenzene-benzoic acid ring in the structure of the related compound 2-[5-(2-fluorophenyl)-3-isobutyl-1H-pyrazol-1-yl]benzoic acid (Sreenivasa et al., 2013) are 53.1 (1)°, 52.1 (1)° and 62.1 (1)°, respectively. Aromatic π–π stacking interactions between the difluorobenzene rings in the title structure [centroid-to-centroid separation = 3.6082 (11) Å] links the molecules parallel to [212] in the crystal structure (Fig 2).

### 2. Experimental

1-(3,5-Difluorophenyl)-4,4,4-trifluorobutane-1,3-dione (0.015 mmol) and (4-fluorophenyl)hydrazine (0.015 mmol) were taken in dry ethanol (14 ml) and the reaction mixture was stirred for 6 h at 348 K under a nitrogen atmosphere. The reaction was monitored by TLC and the excess solvent was removed by vacuum to obtain the crude compound. It was purified by column chromatography using dichloromethane and ethanol (9:1) as eluent.

Yellow prisms suitable for diffraction studies were grown by slow evaporation of the solvent system: dichloromethane and methanol (9:1).

### 3. Refinement

The H atoms were positioned with idealized geometry using a riding model with C—H = 0.93 Å. The isotropic displacement parameters for all H atoms were set to 1.2 times *U*_eq_ of the parent atom.

### Crystal data

**Table:**

C_16_H_8_F_6_N_2_	*F*(000) = 344
*M**_r_* = 342.24	Prism
Triclinic, *P*1	*D*_x_ = 1.650 Mg m^−^^3^
Hall symbol: -P 1	Melting point: 456 K
*a* = 7.2535 (3) Å	Cu *K*α radiation, λ = 1.54178 Å
*b* = 8.6686 (4) Å	Cell parameters from 1234 reflections
*c* = 11.7690 (5) Å	θ = 4.0–64.8°
α = 70.909 (1)°	µ = 1.39 mm^−^^1^
β = 80.139 (1)°	*T* = 293 K
γ = 88.077 (1)°	Prism, yellow
*V* = 688.78 (5) Å^3^	0.39 × 0.35 × 0.29 mm
*Z* = 2	

### Data collection

**Table:**

Bruker APEXII CCD diffractometer	2181 independent reflections
Radiation source: fine-focus sealed tube	2040 reflections with *I* > 2σ(*I*)
Graphite monochromator	*R*_int_ = 0.042
φ and ω scans	θ_max_ = 64.8°, θ_min_ = 4.0°
Absorption correction: multi-scan (*SADABS*; Bruker, 2009)	*h* = −8→8
*T*_min_ = 0.611, *T*_max_ = 0.669	*k* = −9→10
7069 measured reflections	*l* = −13→13

### Refinement

**Table:**

Refinement on *F*^2^	Primary atom site location: structure-invariant direct methods
Least-squares matrix: full	Secondary atom site location: difference Fourier map
*R*[*F*^2^ > 2σ(*F*^2^)] = 0.054	Hydrogen site location: inferred from neighbouring sites
*wR*(*F*^2^) = 0.181	H-atom parameters constrained
*S* = 1.11	*w* = 1/[σ^2^(*F*_o_^2^) + (0.1212*P*)^2^ + 0.5142*P*] where *P* = (*F*_o_^2^ + 2*F*_c_^2^)/3
2181 reflections	(Δ/σ)_max_ < 0.001
217 parameters	Δρ_max_ = 0.45 e Å^−^^3^
0 restraints	Δρ_min_ = −0.48 e Å^−^^3^

### Special details

**Table:**

Geometry. All s.u.'s (except the s.u. in the dihedral angle between two l.s. planes) are estimated using the full covariance matrix. The cell s.u.'s are taken into account individually in the estimation of s.u.'s in distances, angles and torsion angles; correlations between s.u.'s in cell parameters are only used when they are defined by crystal symmetry. An approximate (isotropic) treatment of cell s.u.'s is used for estimating s.u.'s involving l.s. planes.
Refinement. Refinement of *F*^2^ against ALL reflections. The weighted *R*-factor *wR* and goodness of fit *S* are based on *F*^2^, conventional *R*-factors *R* are based on *F*, with *F* set to zero for negative *F*^2^. The threshold expression of *F*^2^ > 2σ(*F*^2^) is used only for calculating *R*-factors(gt) *etc*. and is not relevant to the choice of reflections for refinement. *R*-factors based on *F*^2^ are statistically about twice as large as those based on *F*, and *R*- factors based on ALL data will be even larger.

### Fractional atomic coordinates and isotropic or equivalent isotropic displacement parameters (Å^2^)

**Table:**

	*x*	*y*	*z*	*U*_iso_*/*U*_eq_	
F5	0.4695 (2)	0.16122 (18)	0.61518 (14)	0.0259 (4)	
F4	0.0082 (2)	0.5540 (2)	0.60896 (14)	0.0277 (4)	
F3	0.8754 (2)	1.09529 (19)	0.57955 (14)	0.0311 (4)	
F2	0.8448 (2)	1.0637 (2)	0.77056 (15)	0.0338 (5)	
F6	0.7353 (3)	−0.0365 (2)	1.12881 (15)	0.0368 (5)	
F1	1.0813 (2)	0.9640 (2)	0.68380 (16)	0.0329 (5)	
N2	0.7064 (3)	0.5860 (3)	0.80633 (18)	0.0181 (5)	
C8	0.7111 (3)	0.4265 (3)	0.8934 (2)	0.0178 (6)	
C2	0.5326 (3)	0.3904 (3)	0.6683 (2)	0.0186 (6)	
H2	0.6510	0.3520	0.6815	0.022*	
C10	0.5548 (4)	0.1787 (3)	1.0287 (2)	0.0231 (6)	
H10	0.4464	0.1152	1.0651	0.028*	
C7	0.6008 (3)	0.6408 (3)	0.7159 (2)	0.0178 (5)	
C3	0.4110 (4)	0.3037 (3)	0.6324 (2)	0.0188 (5)	
C14	0.7893 (3)	0.8337 (3)	0.7149 (2)	0.0191 (6)	
C4	0.2343 (3)	0.3540 (3)	0.6116 (2)	0.0196 (6)	
H4	0.1552	0.2928	0.5875	0.024*	
C5	0.1808 (3)	0.5007 (3)	0.6286 (2)	0.0196 (6)	
N1	0.8244 (3)	0.7042 (3)	0.80627 (19)	0.0197 (5)	
C16	0.8973 (3)	0.9877 (3)	0.6872 (2)	0.0222 (6)	
C11	0.7271 (4)	0.1186 (3)	1.0535 (2)	0.0252 (6)	
C6	0.2943 (3)	0.5943 (3)	0.6650 (2)	0.0191 (6)	
H6	0.2529	0.6918	0.6763	0.023*	
C1	0.4720 (3)	0.5386 (3)	0.6844 (2)	0.0174 (5)	
C13	0.8827 (4)	0.3653 (3)	0.9218 (2)	0.0230 (6)	
H13	0.9914	0.4288	0.8860	0.028*	
C15	0.6500 (3)	0.8044 (3)	0.6551 (2)	0.0187 (6)	
H15	0.6018	0.8771	0.5902	0.022*	
C9	0.5463 (3)	0.3358 (3)	0.9483 (2)	0.0207 (6)	
H9	0.4315	0.3799	0.9314	0.025*	
C12	0.8922 (4)	0.2094 (4)	1.0036 (2)	0.0276 (6)	
H12	1.0060	0.1672	1.0243	0.033*	

### Atomic displacement parameters (Å^2^)

**Table:**

	*U*^11^	*U*^22^	*U*^33^	*U*^12^	*U*^13^	*U*^23^
F5	0.0287 (8)	0.0245 (8)	0.0340 (9)	0.0044 (6)	−0.0110 (6)	−0.0196 (7)
F4	0.0148 (7)	0.0429 (10)	0.0349 (9)	0.0067 (6)	−0.0103 (6)	−0.0230 (8)
F3	0.0322 (9)	0.0291 (9)	0.0316 (9)	−0.0068 (7)	−0.0084 (7)	−0.0073 (7)
F2	0.0414 (10)	0.0309 (9)	0.0371 (10)	−0.0063 (7)	−0.0009 (7)	−0.0241 (8)
F6	0.0465 (10)	0.0285 (10)	0.0311 (9)	−0.0012 (8)	−0.0096 (8)	−0.0021 (7)
F1	0.0195 (8)	0.0299 (9)	0.0533 (11)	−0.0036 (6)	−0.0088 (7)	−0.0169 (8)
N2	0.0154 (10)	0.0240 (12)	0.0198 (11)	−0.0024 (8)	−0.0037 (8)	−0.0131 (9)
C8	0.0225 (12)	0.0211 (13)	0.0150 (11)	−0.0015 (10)	−0.0050 (9)	−0.0116 (10)
C2	0.0149 (11)	0.0263 (14)	0.0191 (12)	0.0019 (10)	−0.0053 (9)	−0.0125 (10)
C10	0.0284 (13)	0.0241 (14)	0.0184 (13)	−0.0058 (10)	0.0013 (10)	−0.0113 (11)
C7	0.0135 (11)	0.0257 (13)	0.0197 (12)	0.0001 (9)	−0.0044 (9)	−0.0140 (10)
C3	0.0215 (12)	0.0192 (12)	0.0195 (12)	0.0013 (9)	−0.0033 (9)	−0.0116 (10)
C14	0.0180 (12)	0.0240 (13)	0.0197 (12)	−0.0010 (10)	−0.0027 (9)	−0.0135 (11)
C4	0.0178 (12)	0.0267 (14)	0.0180 (12)	−0.0040 (10)	−0.0036 (9)	−0.0114 (10)
C5	0.0115 (11)	0.0315 (14)	0.0173 (12)	0.0010 (10)	−0.0032 (9)	−0.0098 (10)
N1	0.0175 (10)	0.0232 (12)	0.0227 (11)	−0.0031 (8)	−0.0043 (8)	−0.0127 (9)
C16	0.0202 (12)	0.0267 (14)	0.0246 (14)	0.0011 (10)	−0.0054 (10)	−0.0140 (11)
C11	0.0373 (16)	0.0224 (14)	0.0167 (12)	0.0003 (11)	−0.0061 (11)	−0.0067 (10)
C6	0.0182 (12)	0.0235 (13)	0.0194 (12)	0.0025 (10)	−0.0047 (9)	−0.0116 (10)
C1	0.0157 (11)	0.0241 (13)	0.0159 (12)	−0.0018 (9)	−0.0030 (9)	−0.0108 (10)
C13	0.0214 (13)	0.0283 (14)	0.0219 (13)	−0.0032 (10)	−0.0057 (10)	−0.0103 (11)
C15	0.0182 (12)	0.0215 (13)	0.0208 (12)	0.0022 (10)	−0.0059 (9)	−0.0116 (10)
C9	0.0180 (12)	0.0303 (14)	0.0192 (12)	0.0001 (10)	−0.0018 (9)	−0.0157 (11)
C12	0.0278 (14)	0.0321 (15)	0.0258 (14)	0.0027 (11)	−0.0109 (11)	−0.0105 (12)

### Geometric parameters (Å, º)

**Table:**

F5—C3	1.359 (3)	C7—C15	1.390 (4)
F4—C5	1.351 (3)	C7—C1	1.478 (3)
F3—C16	1.336 (3)	C3—C4	1.376 (4)
F2—C16	1.349 (3)	C14—N1	1.329 (3)
F6—C11	1.353 (3)	C14—C15	1.399 (3)
F1—C16	1.339 (3)	C14—C16	1.483 (3)
N2—N1	1.357 (3)	C4—C5	1.383 (4)
N2—C7	1.367 (3)	C4—H4	0.9300
N2—C8	1.430 (3)	C5—C6	1.383 (3)
C8—C13	1.388 (4)	C11—C12	1.385 (4)
C8—C9	1.390 (4)	C6—C1	1.390 (3)
C2—C3	1.378 (3)	C6—H6	0.9300
C2—C1	1.404 (4)	C13—C12	1.387 (4)
C2—H2	0.9300	C13—H13	0.9300
C10—C11	1.378 (4)	C15—H15	0.9300
C10—C9	1.387 (4)	C9—H9	0.9300
C10—H10	0.9300	C12—H12	0.9300
			
N1—N2—C7	112.0 (2)	F3—C16—F1	107.1 (2)
N1—N2—C8	118.73 (19)	F3—C16—F2	106.1 (2)
C7—N2—C8	129.3 (2)	F1—C16—F2	106.02 (19)
C13—C8—C9	121.0 (2)	F3—C16—C14	111.8 (2)
C13—C8—N2	118.7 (2)	F1—C16—C14	112.6 (2)
C9—C8—N2	120.3 (2)	F2—C16—C14	112.7 (2)
C3—C2—C1	117.9 (2)	F6—C11—C10	118.5 (2)
C3—C2—H2	121.1	F6—C11—C12	118.7 (2)
C1—C2—H2	121.1	C10—C11—C12	122.8 (3)
C11—C10—C9	118.7 (2)	C5—C6—C1	118.2 (2)
C11—C10—H10	120.6	C5—C6—H6	120.9
C9—C10—H10	120.6	C1—C6—H6	120.9
N2—C7—C15	106.9 (2)	C6—C1—C2	120.5 (2)
N2—C7—C1	125.3 (2)	C6—C1—C7	119.4 (2)
C15—C7—C1	127.6 (2)	C2—C1—C7	120.0 (2)
F5—C3—C4	118.0 (2)	C12—C13—C8	120.0 (2)
F5—C3—C2	118.2 (2)	C12—C13—H13	120.0
C4—C3—C2	123.8 (2)	C8—C13—H13	120.0
N1—C14—C15	113.4 (2)	C7—C15—C14	103.6 (2)
N1—C14—C16	118.8 (2)	C7—C15—H15	128.2
C15—C14—C16	127.8 (2)	C14—C15—H15	128.2
C3—C4—C5	116.2 (2)	C10—C9—C8	119.3 (2)
C3—C4—H4	121.9	C10—C9—H9	120.3
C5—C4—H4	121.9	C8—C9—H9	120.3
F4—C5—C4	117.9 (2)	C11—C12—C13	118.0 (2)
F4—C5—C6	118.7 (2)	C11—C12—H12	121.0
C4—C5—C6	123.4 (2)	C13—C12—H12	121.0
C14—N1—N2	104.07 (19)		
			
N1—N2—C8—C13	−37.8 (3)	C9—C10—C11—F6	−177.9 (2)
C7—N2—C8—C13	139.9 (2)	C9—C10—C11—C12	1.4 (4)
N1—N2—C8—C9	142.0 (2)	F4—C5—C6—C1	−179.7 (2)
C7—N2—C8—C9	−40.3 (3)	C4—C5—C6—C1	0.7 (4)
N1—N2—C7—C15	−1.2 (3)	C5—C6—C1—C2	−0.6 (4)
C8—N2—C7—C15	−179.0 (2)	C5—C6—C1—C7	176.2 (2)
N1—N2—C7—C1	174.1 (2)	C3—C2—C1—C6	0.3 (4)
C8—N2—C7—C1	−3.7 (4)	C3—C2—C1—C7	−176.5 (2)
C1—C2—C3—F5	179.2 (2)	N2—C7—C1—C6	134.5 (2)
C1—C2—C3—C4	0.0 (4)	C15—C7—C1—C6	−51.2 (3)
F5—C3—C4—C5	−179.2 (2)	N2—C7—C1—C2	−48.6 (3)
C2—C3—C4—C5	0.0 (4)	C15—C7—C1—C2	125.7 (3)
C3—C4—C5—F4	180.0 (2)	C9—C8—C13—C12	1.8 (4)
C3—C4—C5—C6	−0.4 (4)	N2—C8—C13—C12	−178.4 (2)
C15—C14—N1—N2	0.5 (3)	N2—C7—C15—C14	1.4 (2)
C16—C14—N1—N2	−179.3 (2)	C1—C7—C15—C14	−173.8 (2)
C7—N2—N1—C14	0.5 (2)	N1—C14—C15—C7	−1.2 (3)
C8—N2—N1—C14	178.55 (19)	C16—C14—C15—C7	178.5 (2)
N1—C14—C16—F3	167.2 (2)	C11—C10—C9—C8	1.3 (3)
C15—C14—C16—F3	−12.5 (3)	C13—C8—C9—C10	−2.9 (3)
N1—C14—C16—F1	46.5 (3)	N2—C8—C9—C10	177.3 (2)
C15—C14—C16—F1	−133.1 (3)	F6—C11—C12—C13	176.8 (2)
N1—C14—C16—F2	−73.3 (3)	C10—C11—C12—C13	−2.4 (4)
C15—C14—C16—F2	107.0 (3)	C8—C13—C12—C11	0.8 (4)
